# Whole-Genome Analysis of Porcine Epidemic Diarrhea Virus from Yunnan, China

**DOI:** 10.3390/vetsci11110548

**Published:** 2024-11-06

**Authors:** Runting Zhang, Gefen Yin, Yunhua Wang, Yongneng Li, Xinxian Wang, Junlong Bi, Guishu Yang, Kaixing Qu, Libo Gao

**Affiliations:** 1 College of Animal Veterinary Medicine, Yunnan Agricultural University, Kunming 650201, China; cottenwoolball9@yeah.net (R.Z.); yingefen@ynau.edu.cn (G.Y.); wyhgna@sina.com (Y.W.); yongnengli1008@yeah.net (Y.L.); wangxinxian2024@126.com (X.W.); junlongbi@foxmail.com (J.B.); 1989015@ynau.edu.cn (G.Y.); 2 Academy of Science and Technology, Chuxiong Normal University, Chuxiong 675000, China

**Keywords:** Yunnan province, porcine epidemic diarrhea virus, genetic variation, genome recombination, phylogenetic analysis

## Abstract

Porcine epidemic diarrhea virus (PEDV) is a member of the genus α-coronavirus and causes severe diarrhea in piglets, leading to enormous economic losses in the pig industry. To understand the epidemic variation of PEDV strains in Yunnan province, three PEDV strains (YN2021, YNLP 2022, and YNBS 2022) and one commercially available attenuated vaccine strain (Attenuated AJ1102-R) that were previously isolated were sequenced and compared with the representative PEDV strains. NJ phylogenetic analysis showed that YN2021 strain and Attenuated CV777 strain were clustered into GI-b subtype, while YNLP 2022 and YNBS 2022 belong to GII-b subtype, accompanying ZJCZ4 and Attenuated AJ1102-R. RDP analysis revealed that YNLP 2022 was a genome recombination from both GII-b strain PEDV-7C and GII-a strain YN1, of which the recombination region is in the range nt4994–7605. YNBS 2022 strain was another recombination originated from GII-b subtype strain 17GXZC-1ORF3c and GII-a subtype strain PEDV-CHZ, of which the counterpart is in the range nt16399–22326. The Yunnan strain of PEDV was analyzed for the first time from the whole-genome perspective, and comprehensive analysis showed that the Yunnan strains have high genetic variation. This study may shed new light on the current PEDV infections in Yunnan and pave the way toward further control of PEDV infections.

## Simple Summary

Porcine epidemic diarrhea virus (PEDV) infection causes severe diarrhea in piglets and is potentially lethal, eventually leading to economic losses in the pig industry. At present, the lack of whole-genome information and genetic evolution characteristics of PEDV strains in Yunnan has limited our understanding of PEDV epidemic variation. This is the first time that we have identified the complete genome of the Yunnan strain and explored its genetic evolutionary characteristics. This study may shed new light on the current PEDV infections and pave the way toward further control of PEDV infections in Yunnan.

## 1. Introduction

Porcine epidemic diarrhea (PED) is a highly contagious intestinal disease caused by porcine epidemic diarrhea virus (PEDV), which is infectious to all swine productive stages and has high mortality in newborn piglets [1], with 100% morbidity and 80–100% mortality in neonatal piglets [2,3]. PED was initially discovered in the UK in 1971 [4] and then spread to other European countries, where it occasionally broke out in the latter half of the 20th century [5,6]. There have been occasional outbreaks of PEDV in the swine population in China and around the world. However, a more widespread outbreak of PEDV occurred in southern China in late 2010 and quickly spread to other provinces [7,8]. The identification and sequencing of PEDV strains in this outbreak revealed the emergence of a new variant of PEDV, differentiated from classic strains such as CV777 [9], contributing to the high diversity of PEDV variants in China [10,11]. Obviously, the commercially available CV777 inactivated vaccine and the DR13 attenuated vaccine do not provide effective protection against PEDV variants, making prevention and control difficult to achieve [12,13].

PEDV belongs to the genus *Alphacoronavirus* within the family *Coronaviridae* and is an enveloped single-stranded positive-sense RNA virus [14]. It features a characteristic nested crown and has a genome size of approximately 28 kb [15]. The PEDV genome contains seven overlapping open reading frames (ORF), which encode the replicase (ORF1a, 1b), nonstructural proteins, the accessory protein ORF3, the spike (S), the envelope (E), the membrane (M), and the nucleocapsid (N) structural proteins [16]. The PEDV genome is arranged as 5′ UTR-ORF1-S-ORF3-E-M-N-3′ UTR [17].

PEDVs can be categorized into two major groups: GI and GII [18]. GI includes classical strains, represented by CV777, such as DR13, SM98, and other early strains mainly found in Europe and Asia [19]. All the PEDVs isolated in China before 2010 belonged to GI. The main pandemic strain currently circulating in China fell into GII since 2010, which can be further divided into GII-a, GII-b, and GII-c. GII-a consists of mutant strains mainly from China, the United States, Germany, and France, such as SC-YB73, GDS28 (common in Asia), and Colorado and PC21A (common in North America). GII-b strains predominantly originated from China and other Asian countries, and these strains are generally early strains or cell-adapted attenuated strains caused by S-INDEL [14]. GI-b is less virulent than GI-a, while GII-b strains include Attenuated CV777 and Attenuated DR13. However, SH strain belonged to GI-b, having high virulence with a unique 12-aa deletion (aa 399–410) including the antigenic epitope NEP-1C9 (aa 398–406) of the N protein [20], suggesting that amino acid (aa) substitution in the N protein could be a pattern of GII-b subtype evolution. GII-c is an S-INDEL strain produced by the recombination of GI-a and GII-a types and structural variants of the S gene [18,21]. The less virulent GII-c strains, consisting of OH851, CH/HNQX-3/14, and ZL29, are mainly transmitted and prevalent in Europe, with a minority coming from the United States and Asia [2]. The S-INDEL strain, in comparison to GI/GII strains, has multiple insertions and deletions in the S gene, firstly identified in the United States in 2014, represented by strain OH851, and later spread to European countries such as Germany, France, Hungary, Spain, Belgium, Poland, Slovenia, Romania, Austria, and Italy [22,23,24]. The earliest reported S-INDEL strain in China was the ZL29 strain from Hubei in 2015. Compared with the GI strains, the PEDV S-INDEL strains differ from the PEDV GII strains because they were defined by 2 aa insertion (161–162 aa) and 5 aa deletions (at positions 59–62 aa and 140 aa), while GII was also classified as non-S-INDEL [25]. It has been confirmed that PEDV mutated and is prevailing in China, and the traditional vaccine PEDV CV777 cannot completely protect against highly pathogenic variants [7,26,27]. 

In today’s interconnected world, animal coronaviruses such as PEDVs have many opportunities to spread between countries and even continents, and the genetic diversity of PEDVs on a global scale has quickly increased. China has the largest pig population worldwide, with over 450 million pigs [28]. Such a large pig population probably increases the likelihood of new virus strains emerging. It is crucial to understand the molecular biological characteristics of PEDV epidemic strains in each region. Yunnan province in China, which shares longer borders with Myanmar, Laos, and Vietnam, has a unique geography that might complicate virus evolution due to its distinctive environment. However, there are limited reports on the whole genome of Yunnan PEDV epidemic strains. Herein, we conducted analysis involving the molecular characteristics, genetic evolution, and genome recombination of three Yunnan PEDV epidemic strains and commercial vaccine strains to provide more basic information for the prevention and control of PEDVs.

## 2. Materials and Methods

### 2.1. Sample Collection and PEDV Detection

A total of 219 samples were collected from the small intestine tissues, diarrhea feces, or blood from suckling piglets in Yunnan province, China, between February 2021 and December 2023. The piglets had symptoms including vomiting, severe watery diarrhea, and dehydration and were therefore suspected to be infected with PEDV. The diarrhea feces were obtained with medical swabs and resuspended in 1.0 mL 0.9% sodium chloride solution in 1.5 mL Eppendorf tubes. After centrifugation at 10,000× *g* for 10 min, 200 μL supernatants were transferred into new tubes for −80 °C storage until RNA extraction. A commercially available vaccine containing Attenuated AJ1102-R was purchased from Wuhan Keqian Biological Co., Ltd. (Wuhan, China).

Total RNAs were extracted from the collected samples according to the instructions of the RNA extraction kit (RNAiso Plus, Cat. No. AA6702-1, TaKaRa), followed by reverse transcription (EasyScript RT/RI Enzyme Mix, Cat. No. K21105, TransGen Biotech, Beijing, China) and PCR (2 × TransHiFi Super Mix, Cat. No. L10602, TransGen Biotech). All the 219 samples were subjected to reverse transcription polymerase chain reaction (RT-PCR) to identify whether the piglets were PEDV-infected. A primer pair (forward primer 5′-TTTATTCTGTCACGCCATGT-3′ and reverse primer 5′-CCAGATTTACARACACCTATG-3′) was designed according to GenBank Accession No. AF353511.1 to amplify a partial region of S gene with a length of 199 bp. The PCR program consisted of an initial denaturation at 94 °C for 5 min, followed by 25 amplification cycles of denaturation at 94 °C for 1 min, annealing at 53 °C for 1 min, and extension at 72 °C for 90 s, with a final extension step at 72 °C for 7 min.

### 2.2. Sequence Assembly and Alignment of Whole Genome

The complete genome of PEDV was amplified using universal primers (Table S1), which were also designed according to the reference sequence AF353511.1. The PCR products were subjected to 1.5% agarose gel electrophoresis for detecting the presence of PEDV gene fragments by visualization under ultraviolet light after staining with 1.0 μg/mL ethidium bromide (EB). The positive PCR products were electrophoresed on 3.0% agarose gel for the purification of amplified products according to the instructions (Bio Teke Corporation, Beijing, China). The purified PCR products were ligated into pMD18-T vectors (Sangon Biological Engineering Co., Ltd., Shanghai, China). The plasmids were then transformed into *E. coli* DH5α cells (TaKaRa Biotech Co. Ltd., Dalian, China) for 16–18 h culture, followed by plasmid DNA extraction and sequencing (Sangon Biological Engineering Co., Ltd., Shanghai, China).

SeqMan in DNAStar 6.0 (DNAStar Inc., Madison, WI, USA) was used to splice the sequence, check the original sequence peak map and base correspondence, and correct the abnormal base. The complete sequences were identified by comparison with PEDV genome as references from GenBank database, Accession No. AF353511.1 (CV777). The sequence was resequenced; if the base of a site was difficult to determine, the primer was partially sheared to preserve the spliced sequence after determining the correct base.

### 2.3. Database Accession Numbers

To analyze the genetic evolution, genome sequence, amino acid variation of key genes, and the recombination events of Yunnan PEDV strains, the sequences of 69 representatives of PEDV strains (including 57 complete genomes, 9 complete S genes, and 2 complete ORF3 genes) were downloaded from GenBank. The selection of reference strains was based on the principle of including the classical strains and different genotypic PEDV strains [29]. The reference strain information is shown in Table S2. The pathogenic PEDVs YN2021, YNLP 2022, YNBS 2022, and Attenuated AJ1102-R strain used in this study were previously isolated in our lab. And the Attenuated AJ1102-R, a commercially available vaccine, was purchased from Wuhan Keqian Biological Co., Ltd.

### 2.4. Phylogenetic Analysis of S Gene and PEDV Genome

Using MegAlign 6.1 software, the complete nucleotide sequences and deduced amino acid residues of the S gene of 4 isolates were aligned with 57 representative isolates retrieved from the NCBI. The homologies of PEDV genome sequences and S gene were calculated, and the homology comparisons were plotted using ChiPlot online. Multiple sequence alignments between 57 reference sequences and the whole genome obtained were performed using MAFFT method in BioAider V1.423 [30]. The genetic distances were calculated, and the phylogenetic tree was constructed by Neighbor-Joining (NJ) method of MEGA11 and the maximum composite likelihood method. The branch confidence was checked by bootstrap method with a setting of 1000 repeats.

### 2.5. Genetic Recombination Analysis

Potential recombination events were detected using RDP4 (4.70) software, including RDP, GENECONV, BootScan, MaxChi, Chimaera, SiScan, and 3seq [31]; recombination events were considered significant when at least 5 out of 7 algorisms showed *p* < 0.001. The RDP window size was set to 30 bp, and other parameters were set to the default values. The above methods were further validated and confirmed for the presence of genome recombination and break sites using BootScan in Simplot software, with the BootScan window size set to 400 bp, the gap size set to 40 bp, and other parameters set to default values.

## 3. Results

### 3.1. Epidemiology of PEDV in Yunnan from February 2021 to December 2023

A total of 219 samples were collected from Yunnan province during the period from February 2021 to December 2023, and RT-PCR analysis showed that 21.46% (47/219) of the detected samples were identified as PEDV-positive (Table 1). 

### 3.2. Genome Information of Yunnan PEDV Strains

**Figure 1:**
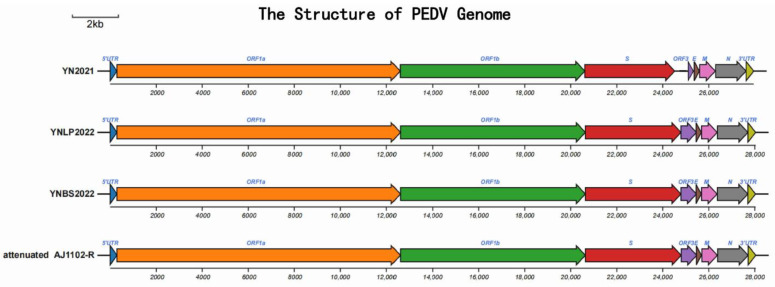
Schematic diagram of PEDV YN2021, YNLP 2022, YNBS2022, and Attenuated AJ1102-R strain of genome structure. Non-structural proteins, including ORF1a (orange) and ORF1B (green); structural proteins, including spike (S, red), ORF3 (purple), envelope (E, brick red), membrane (M, pink), and nucleocapsid (N, grey) proteins; accessory proteins.

After cloning, sequencing, and assembling, the sequences of three epidemic strains from Yunnan and one commercial attenuated vaccine strain were obtained successfully, of which the full genome lengths of YN2021 (Kunming city), YNLP 2022 (Lanping county), YNBS 2022 (Baoshan city), and the Attenuated AJ1102-R were 27,953 bp, 28036 bp, 20,831 bp, and 28,042 bp, respectively. The typical feature of the PEDV genome is the 5′ UTR-ORF1A/1b-S-ORF3-E-M-N-3′ UTR (Table 2, Figure 1), consistent with Brian’s research [17]. Four PEDV strains were submitted to GenBank with the following accession numbers: YN2021 (OQ437175), YNLP 2022 (OQ437174), YNBS 2022 (OP972835), and AJ1102-R (OQ589489).

### 3.3. Phylogenetic Analysis of PEDV Genome

Genome-wide nucleotide homology analysis showed that YN2021 and Attenuated CV777 belong to GI-b subtype, with the highest homology (99.8%), while YNBS 2022 and Attenuated AJ1102-R belong to GII-b subclade. YNLP 2022 and CHGD-01 clustered into the same clade, with higher homology (97.7%) (Figure S1).

**Figure 2:**
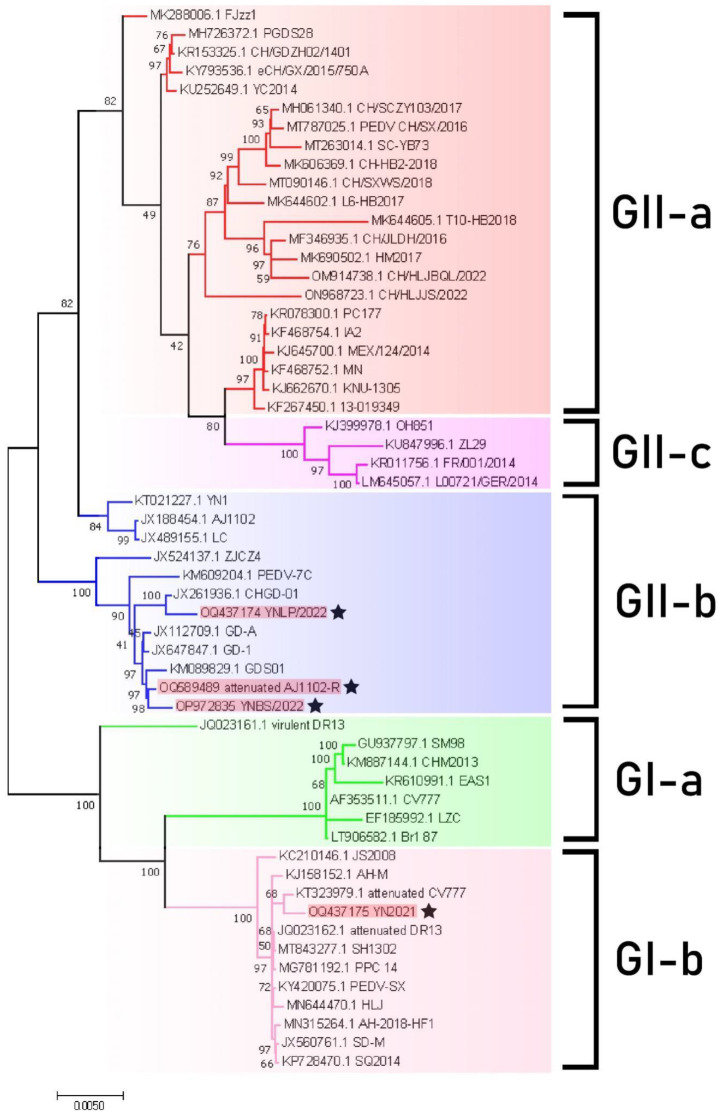
NJ phylogenetic tree of the genomes of PEDV strain, including three isolates in this study from Yunnan and Attenuated AJ1102-R as the reference (four strains highlighted in red and marked with stars). A phylogenetic tree was constructed by maximum composite likelihood method and NJ method with > 40% support from 1000 replicates using MEGA 11. The numbers on each branch indicate the bootstrap value.

**Figure 3:**
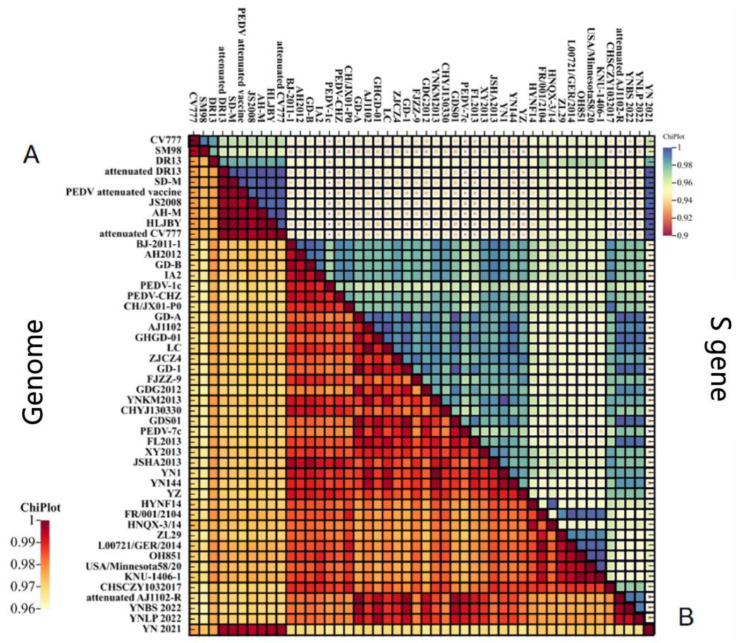
Homology analyses of the complete genomes of PEDV strains and S gene. (**A**) Heatmap of sequence homology of whole genome; (**B**) heatmap of sequence homology of S gene.

The NJ phylogenetic tree was constructed by combining 57 reference strains (including 4 complete genomes obtained in this study) using MEGA 11 (Figure 2, Table S2). The phylogenetic tree depicted two major clusters—GI and GII—while GI was subdivided into GI-a and GI-b, and GII was subdivided into GII-a, GII-b, and GII-c. According to the phylogenetic tree, YN2021, Attenuated CV777, and Attenuated DR13 were clustered into the GI-b subclade. YNLP 2022, YNBS 2022, and Attenuated AJ1102-R formed a separate branch, with the representative strain ZJCZ4 belonging to GII-b type. Meanwhile, YNLP 2022, GHGD-01, and Attenuated AJ1102-R clustered into the same branch, indicating a closer relationship (Figure S2 and Figure 3).

### 3.4. Genetic Variation of S Gene

**Figure 4:**
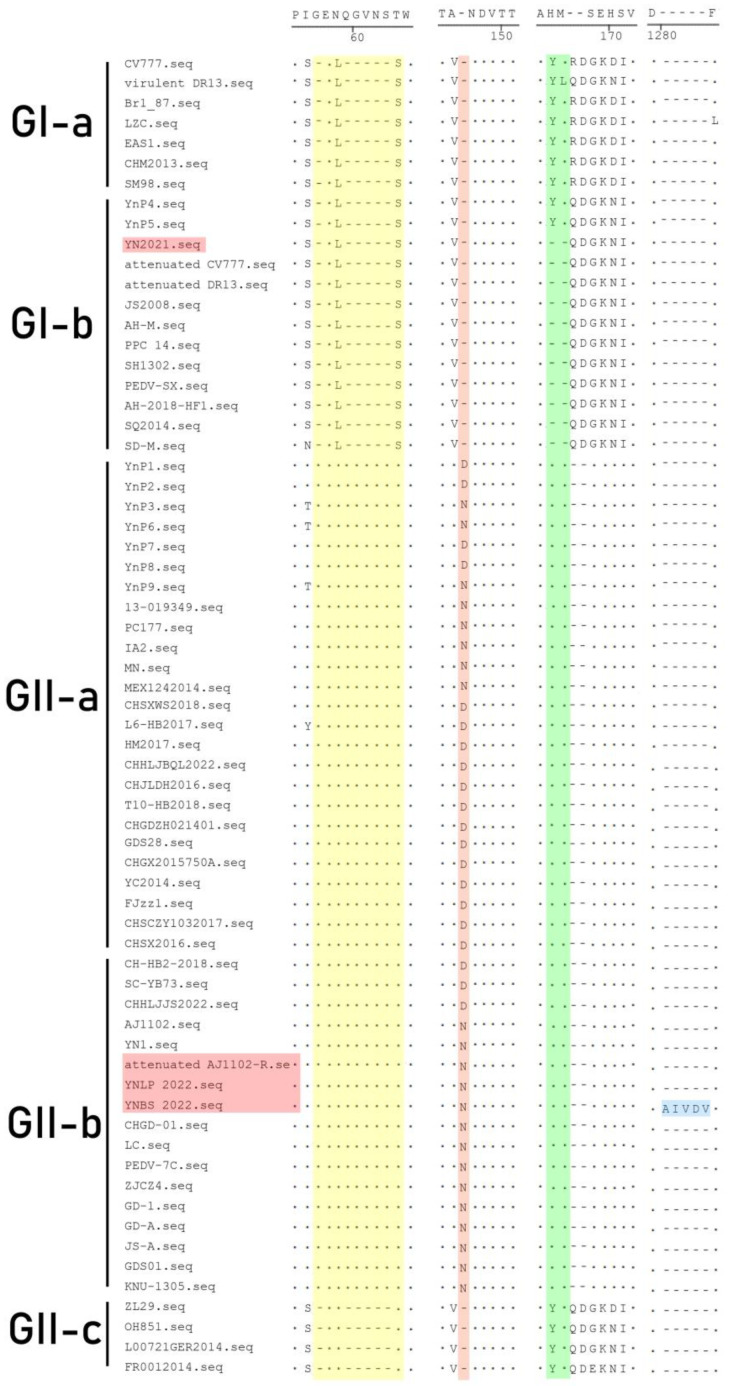
Amino acid variation analysis of PEDV S protein. The dashes (-) indicate the deletion, the dots (·) indicate the same amino acid with the consensus sequence. The Yunnan strains in this study are shown in red boxes. The homology between sequenced strains (names highlighted in red) and reference strains are shown separately in yellow (^56^G, ^59^QGVNS^63^), orange (^146^N/D), and green (position 164, 165 aa) box. The insertion residues (^1280^AIVDV^1284^) are shown in blue.

Nucleotide homologies of PEDV S gene showed significant differences in the heatmap (Figure S2 and Figure 3). Compared with the S gene of the classical strain CV777, GII-a and GII-b strains were usually characterized by amino acid (aa) insertions at positions 56 aa, 59–63 aa, and 146 aa (^56^G, ^59^QGVNS^63^, ^146^N/D) and substitution of L and N at position 58. Compared with classical CV777 and DR13, Attenuated CV777 and Attenuated DR13 had two aa deletions at position 164 and 165 aa (Figure 4). The genetic variations of the YN2021 strain were the same as CV777, while YNLP 2022 and YNBS 2022 were consistent with AJ1102, indicating that YN2021 belongs to a classical epidemic strain, while YNLP 2022 and YNBS 2022 belong to the prevalent strains. YNBS 2022 had five unique amino acid mutations (Table 3), and YN2021 had one mutation at position 577 aa from Q to R, while YNLP belonged to the GII type but had the same amino acid pattern as the classical strain, which was D at position 1249 aa, of which YNP1 to YNP9 were identified previously [32].

**Figure 5:**
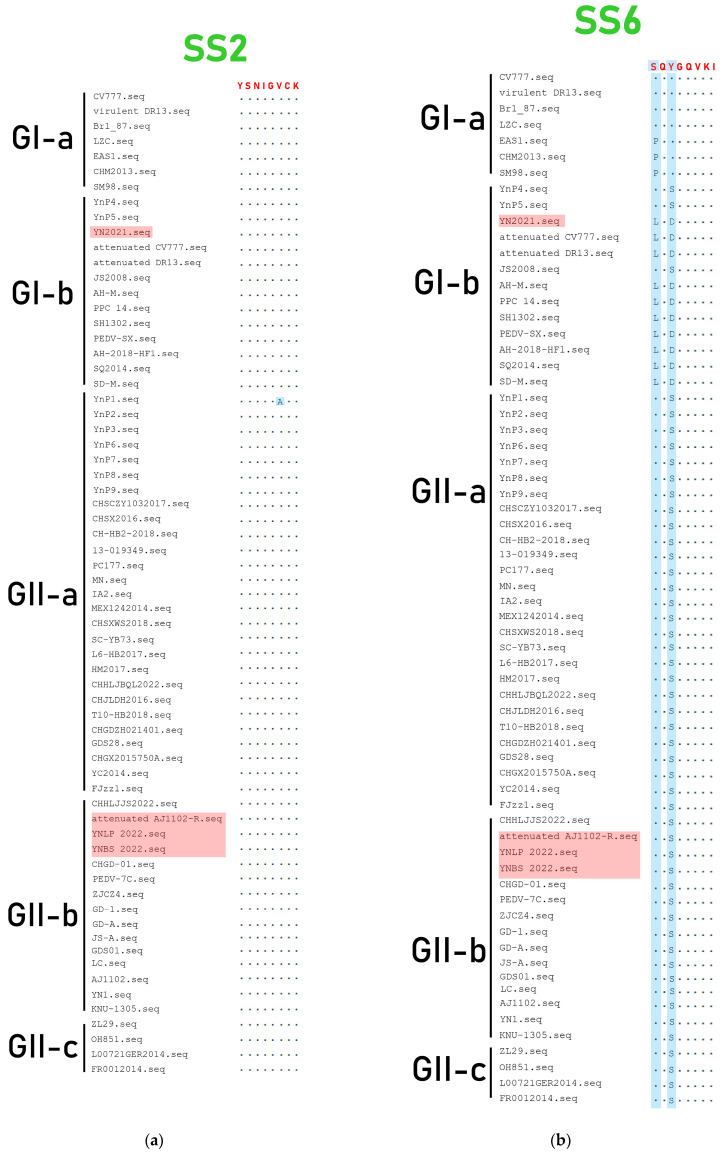

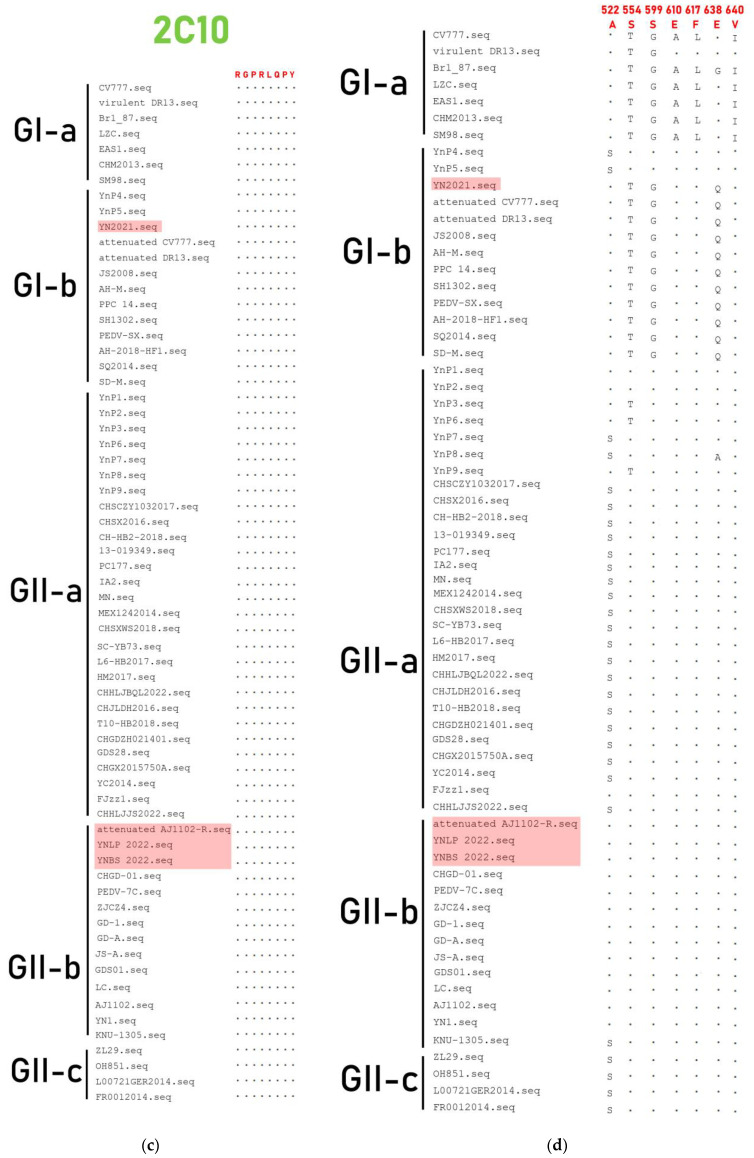
Amino acid mutation of SS epitope of PEDV S protein. The Yunnan strains in this study were showed in red boxes. The dots (·) indicate the same amino acid with the consensus sequence. (**a**) Amino acid mutation of SS2 epitope of PEDV S protein. The Yunnan isolate YNP1 mutated from V to A at position 6 compared with the consensus sequence (highlighted in blue). (**b**) Amino acid mutation of SS6 epitope of PEDV S proteins. Comparing with CV777 and DR13, Attenuated CV777 and Attenuated DR13 mutated from S to L in the first amino acid of SS6 epitope, the third amino acid mutated from S to D (highlighted in blue). (**c**) Amino acid mutation of 2C10 epitope of PEDV S protein. (**d**) Amino acid variation of COE epitope of PEDV S proteins.

Based on the amino acid differences of the S genes between Yunnan PEDV and CV777 strain, four neutralizing epitopes of the Yunnan PEDV strains (SS2, SS6, COE, and 2C10) were analyzed in this study. The results showed that compared with other strains, only the Yunnan isolate YNP1 has A at position 6 (Figure 5a). The SS6 epitope mutations of the Yunnan endemic strains PEDV, YNLP 2022, and YNBS 2022 were the same as the vaccine stain Attenuated AJ1102-R, along with the GII strains shown in Figure 5b. Antigenic epitopes of PEDV strains 2C10 were usually conserved among the strains [33], and no amino acid mutation in this antigenic epitope was found in the four strains in this study (Figure 5c). Analysis of amino acid mutations in the COE epitope of the four strains showed that the most common amino acid mutations occurred at positions 522, 554, 599, 610, 617, 638, and 640 aa, especially at position 638 aa, which the amino acids could mutate from E to G, Q, V, and A, compared with strain CV777 (Figure 5d).

### 3.5. Genetic Variation of ORF3 Gene

**Figure 6:**
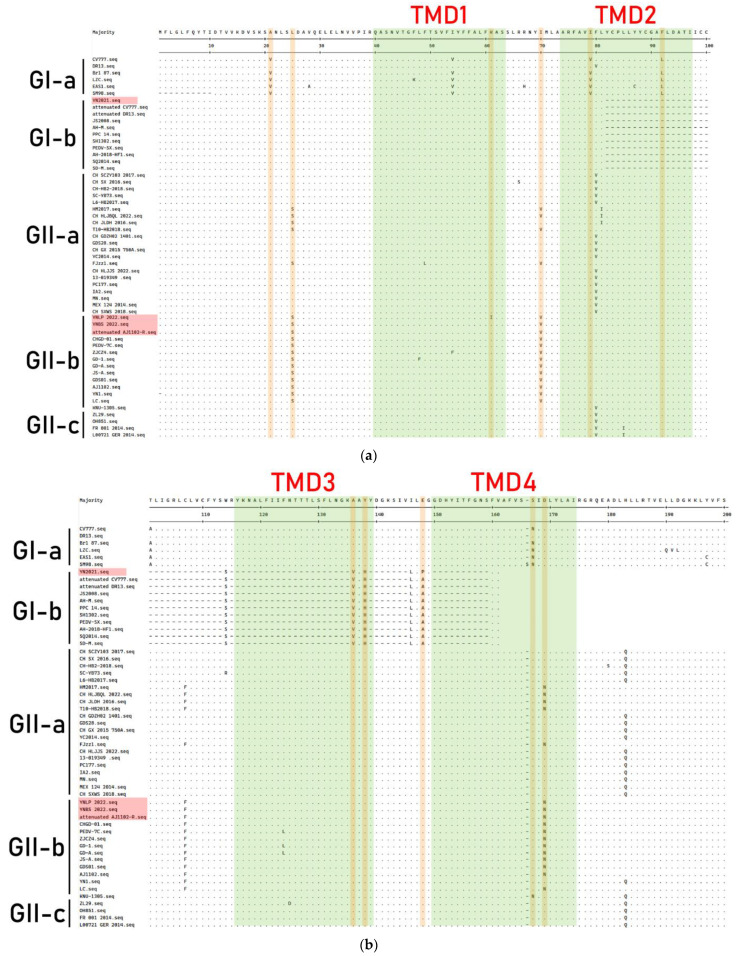

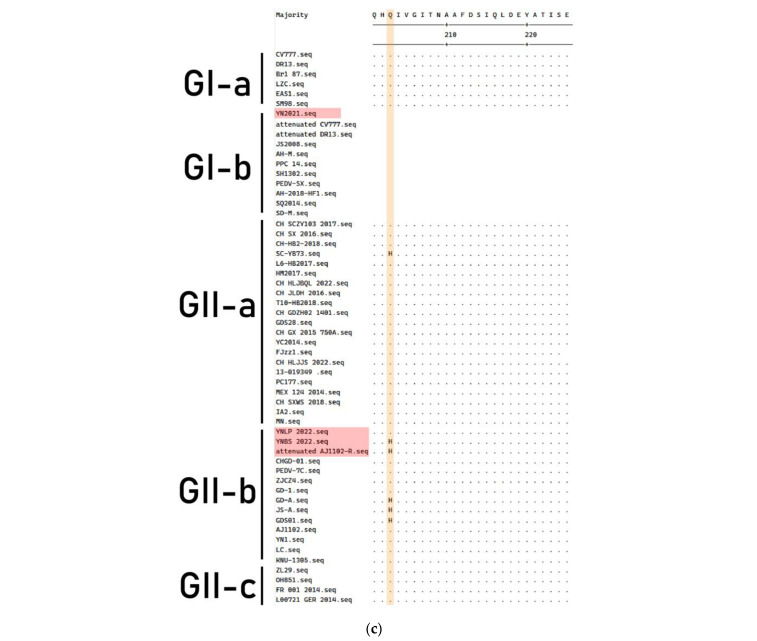
Comparison of amino acid residues of PEDV ORF3 protein. The Yunnan strains in this study were showed in red boxes. The dashes (-) indicate the deletion, the dots (·) indicate the same amino acid with the consensus sequence. (**a**–**c**) The amino acid mutations are shown in yellow boxes, and the transmembrane domain are shown in green boxes.

The amino acid sequences of the ORF3 gene of PEDV strain were aligned (Figure 6). The results showed that compared with the classical strain CV777, the attenuated classical vaccine strain had many amino acid deletions and mutations. The YN2021 strain lacked the TMD4 transmembrane domain (133 aa residues deletion) and had amino acid mutations in the other three transmembrane domains (TMD1, TMD2, and TMD3). The YNLP 2022, YNBS 2022, and Attenuated AJ1102-R strains all had amino acid mutations in TMD1, TMD2, and TMD4. The TMD3 transmembrane domain was relatively conserved and no amino acid variations were found. The four PEDV strains shared two identical amino acid mutations (^V^21^A^ and ^V^79^I^) in the ORF3 gene. The same amino acid mutations (^L^25^S^, ^I^70^V^, ^L^92^F^, ^N^167^S^, and ^D^169^N^) were found and shared at positions 25, 70, 92, 167, and 169 aa in the YNLP 2022, YNBS 2022, and Attenuated AJ1102-R strains. Both the YNBS 2022 and Attenuated AJ1102-R strains had one mutation (^Q^203^H^) at position 203 aa, while YN2021 mutated from E to P at position 148 aa, and YNLP 2022 mutated from K to I at position 61 aa.

### 3.6. Genetic Variation of M Gene

**Figure 7:**
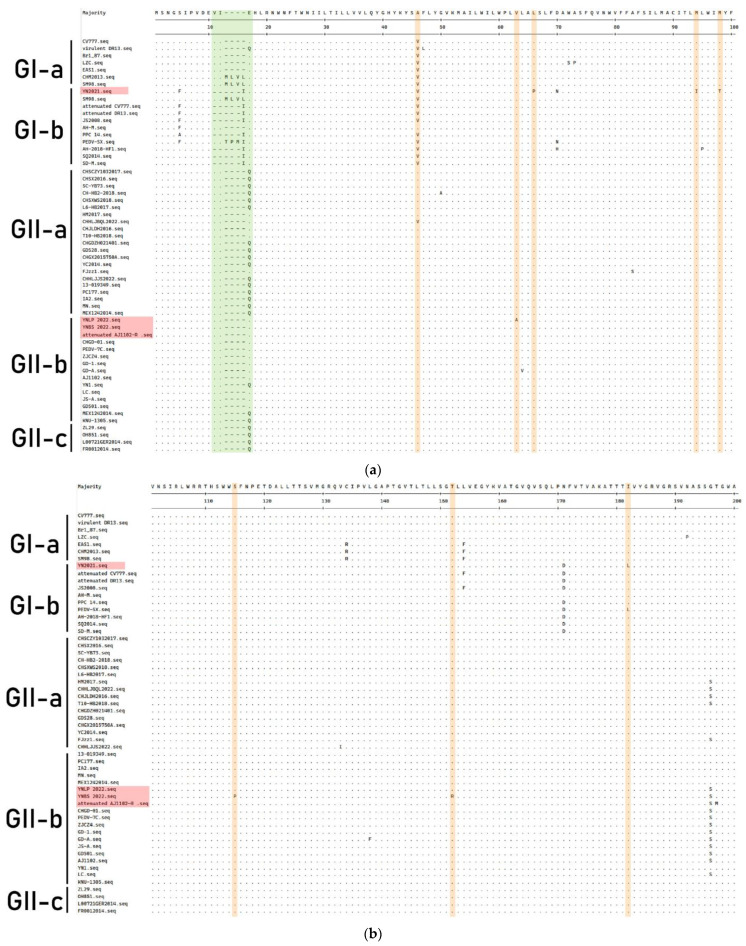

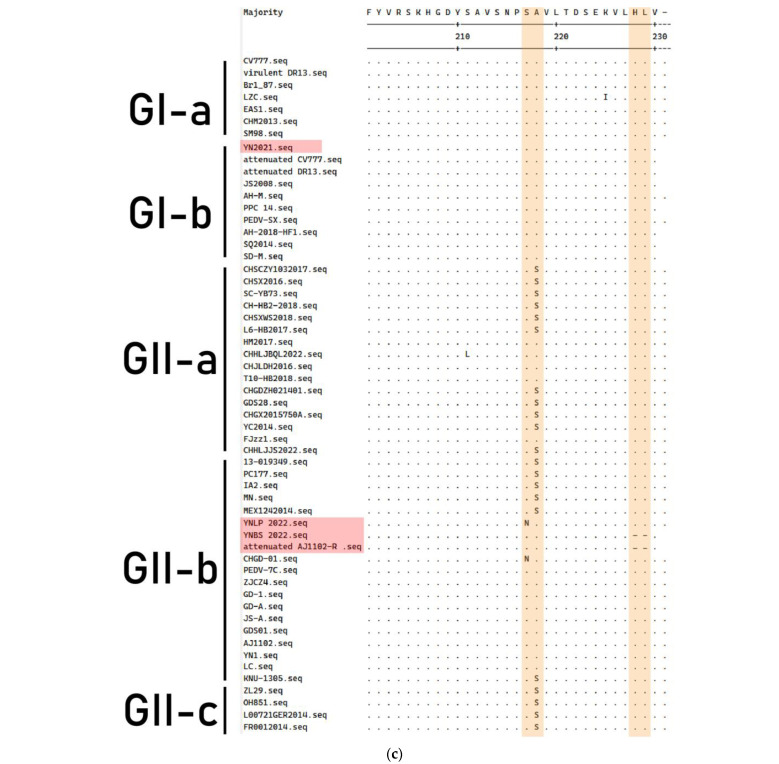
Comparisons of M amino acid residues among PEDV genotypes. The Yunnan strains in this study were showed in red boxes. The dashes (-) indicate the deletion, the dots (·) indicate the same amino acid with the consensus sequence. (**a**–**c**) The yellow box shows the amino acid mutation site, and the green box shows the transmembrane domain.

The amino acid sequence of the M protein of PEDV strain is shown in Figure 7. Compared with the CV777 strain, the YN2021 strain had two aa deletions (^11^VI^12^), one aa insertion (^16^I) at position 11–12 aa, and four aa mutations (^L^66^P^, ^M^94^I^, ^M^98^T^, and ^I^182^L^) found in the M protein. YNLP 2022 underwent two aa mutations (^V^63^A^ and ^S^217^N^), while the latter was the same as Attenuated AJ1102-R. Two aa mutations (^S^115^P^ and ^T^152^R^) and two aa deletions (^228^HL^229^) were found in the YNBS 2022 strain, the same as Attenuated AJ1102.

### 3.7. Genetic Variation of N Protein

**Figure 8:**
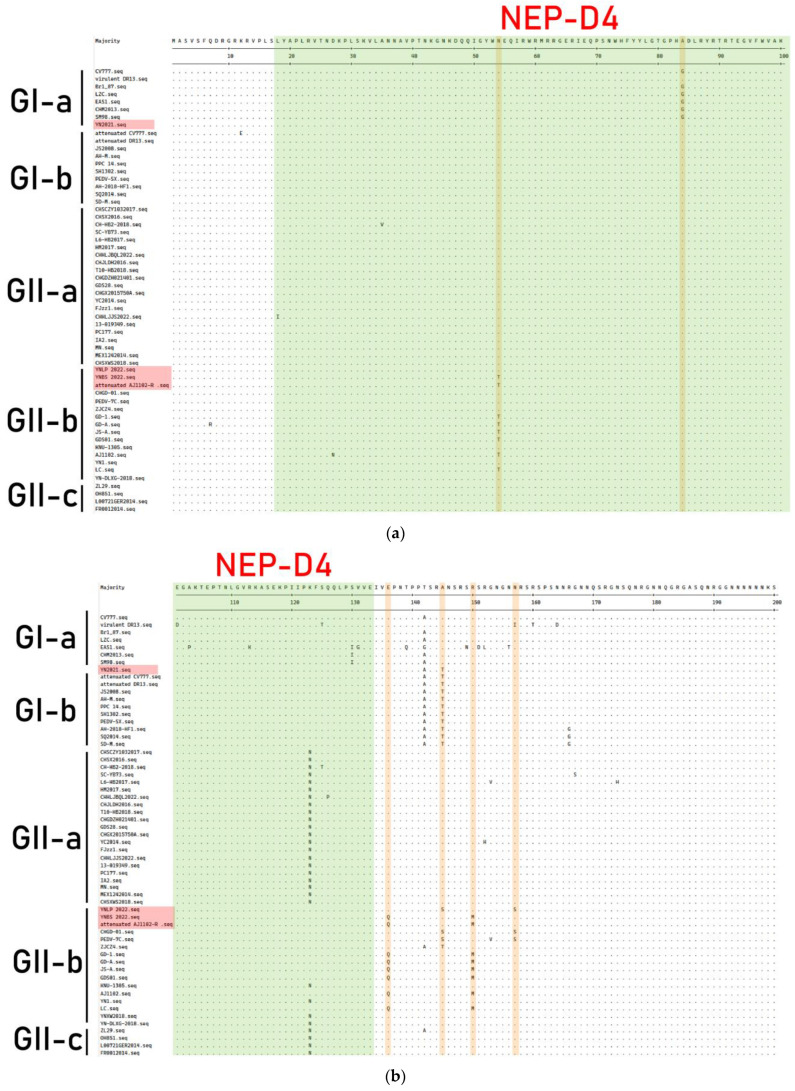

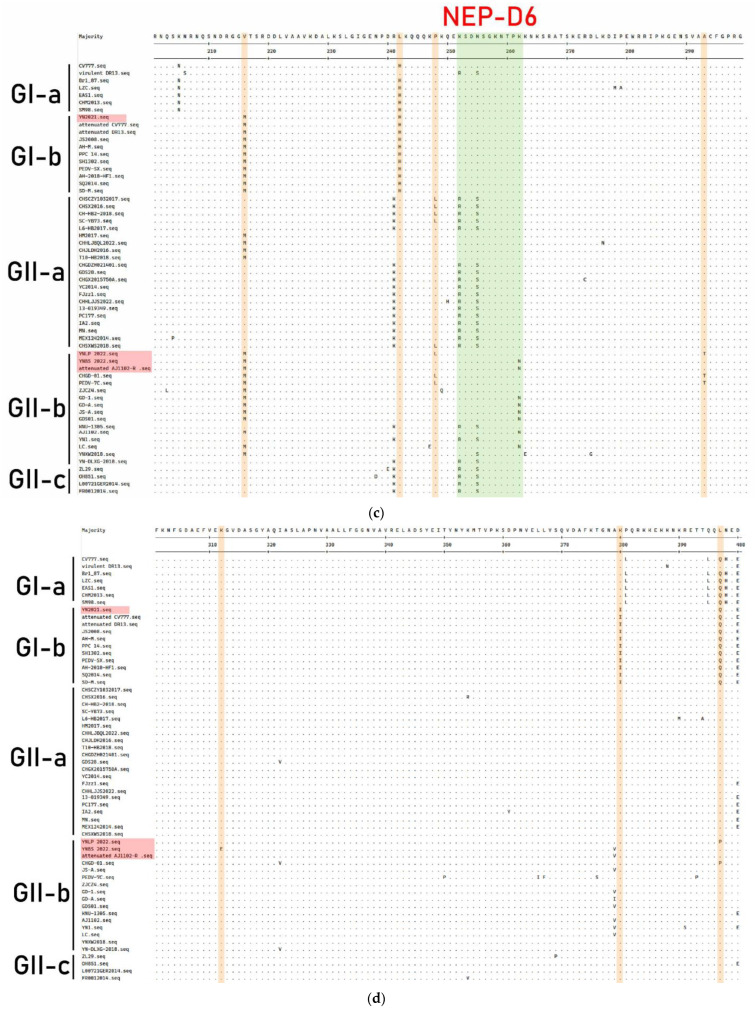
Comparison of amino acid residues of N proteins among PEDV genotypes. The Yunnan strains in this study were showed in red boxes. The dots (·) indicate the same amino acid with the consensus sequence. (**a**–**d**) The amino acid mutations are shown in yellow, and the antigen epitopes are shown in green.

The amino acid sequence of the N gene of the PEDV strain obtained in this study was compared with PEDV strains of different genotypes (Figure 8); the YN2021, YNLP 2022, YNBS 2022, and Attenuated AJ1102-R strains all shared two aa mutations at positions 84 (^G^84^A^) and 216 (^V^216^M^). YN2021 had mutated from A to T at position 145 aa and from K to I at position 380 aa. YNLP 2022 had five aa mutations at positions 145, 157, 248, 293, and 397 aa (^A^145^S^, ^N^157^S^, ^P^248^L^, ^A^293^T^, and ^Q^397^P^). The NEP-D4 and D6 regions of YNBS 2022 strain have two aa variants in ^N^54^T^ and ^K^262^N^, while the other four aa mutations occurred at positions ^E^136^Q^, ^R^150^M^, ^K^312^E^, and ^A^379^V^ outside of NEP-D4/6, respectively. The variation site of the Attenuated AJ1102-R strain was similar to those of the YNBS 2022 strain.

### 3.8. Genome-Wide Recombination Analysis of PEDV

To further analyze the potential recombination in the YN2021, YNLP 2022, and YNBS 2022 strains since prevailing, RDP4 software was used to perform recombination analysis for the whole PEDV genomes. Seven different detection methods of RDP4 (RDP, GENECONV, BootScan, Maxchi, Chimaera, SiScan, and 3SEQ) were simultaneously used to predict YN2021, YNLP 2022, and YNBS 2022, obtaining the *P*-value of each detection method for recombination phenomena and further verifying the likelihood of recombination events by Simplot software. The YNLP 2022 and YNBS 2022 strains were found to infer genome recombination, while only Maxchi and 3seq showed positive recombination signals for the YN2021 strain that the YN2021 strain was excluded. 

**Figure 9:**
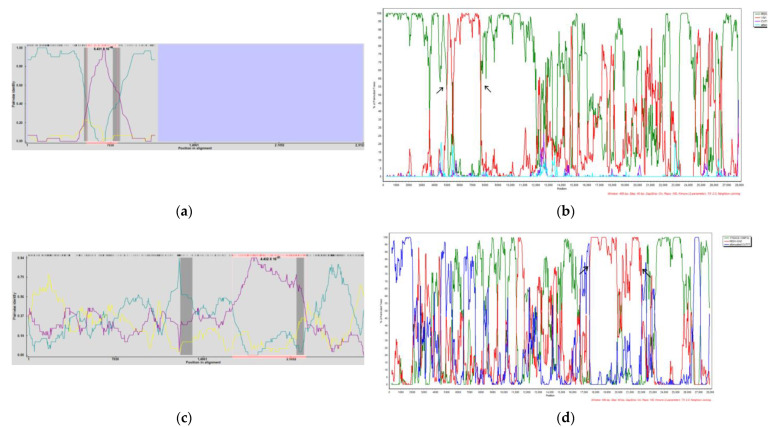
The recombination events of PEDV strains YNLP 2022 and YNBS 2022 identified by BootScan and Simplot analyses. YNLP 2022 (**a**,**b**) and YNBS 2022 (**c**,**d**) recombination events were inferred using RDP, GENECONV, BootScan, Maxchi, Chimaera, SiScan, and 3seq methods. (**a**) YNLP 2022 recombination event, the pairwise identity between each sequence is shown in different color; PEDV 7C and YNLP2022 (green), YN1 and YNLP2022 (purple), PEDV 7C and YN1 (yellow), pink-highlighted region were nt4994-7605, corresponding to the black arrows in (**b**). (**c**) YNBS 2022 recombination event, the pairwise identity between each sequence is shown in different color; 17GXCZ-1ORF3c and YNBS2022 (green), PEDV-CHZ and YNBS2022 (purple), 17GXCZ-1ORF3c and PEDV-CHZ (yellow), pink-highlighted region were nt16399-22326, corresponding to the black arrows in (**d**).

Strikingly, YNLP 2022 was regarded as a recombination originating from the PEDV-7C strain (Jiangsu, China) (major parent) and YN1 strain (Hubei, China) (minor parent), with a breakpoint located at nt4994–7605. Except BootScan, YNBS 2022 had a positive recombination signal (Figure 9c,d, Table 4) to be regarded as a recombination originating from the 17GXCZ-1ORF3c (Guangxi, China) (major parent) and PEDV-CHZ strains (Jiangsu, China) (minor parent), with the breakpoint located at nt16399–22326.

## 4. Discussion

Originally identified as being from the United Kingdom, PEDV is now a long-term global pathogen. The PEDV pandemic was effectively controlled with the widespread use of early-stage Chinese-developed inactivated and attenuated vaccines based on CV777 in China before 2010. However, since 2010, the traditional vaccine for PEDV is not completely effective against highly pathogenic variants [7,20,27]. The highly pathogenic variants had emerged in many countries worldwide, posing a huge economic threat to the global pig industry, while the CV777 vaccine failed to provide complete protection against highly pathogenic variants of PEDV [34,35,36]. It has been confirmed through investigations that many variants of PEDV have occurred in China. These variants have higher virulence and can cause clinical signs in pigs of all ages, resulting in 80–100% mortality in piglets [26,37]. As a result, understanding the prevalence and variation characteristics of the PEDV genome is of great significance for PEDV epidemiology. In 2016, Song et al. reported the prevalent PEDV in certain areas of Yunnan and sequenced the N gene, which revealed that the similarity between six Yunnan PEDV strains and the classical strain CV777 was only 95.2–95.5% [38]. Additionally, we published the S genes of nine PEDV strains prevalent in Yunnan [32], showing that the genotypes of Yunnan PEDV strains were diverse. But there is no report on the whole genome analysis of PEDV in Yunnan Province. In this study, four complete PEDV genomes were deposited in GenBank—three prevalent PEDV strains and the commercially available attenuated vaccine strain AJ1102-R, of which three Yunnan PEDV strains exhibited different whole-genome lengths (27,953 bp to YN2021, 28,036 bp to YNLP 2022, 28,031 bp to 28,031 bp), indicating genome-level differences between the Yunnan prevalent strains and the vaccine strain.

Through analysis of the nucleotide homology and phylogenetic tree of the whole genome, it was found that the YN2021 strain and Attenuated CV777 clustered into the same branch, with the highest homology of 99.8%, belonging to the GI-b subclade. The YNLP 2022 strain and Guangdong, China, isolate CHGD-01 fell into the GII-b subclade, with the highest homology of 97.7%. The YNBS 2022 strain and the attenuated vaccine strain Attenuated AJ1102-R clustered together, belonging to the GII-b subclade.

S protein interacts with specific host cell receptors to mediate viral binding and entry and is closely related to syncytial formation and induction of neutralizing antibodies. The S protein of GI and GII strains were separately different from the N-terminal domain [39], of which the most typical difference is that the S protein of GII strains have 11 aa mutations (^I^116^T^, ^I^356^T^, ^E^365^Q^, ^T^549^S^, ^G^594^S^, ^N^724^S^, ^A^959^V^, ^S^1044^A^, ^G^1173^D^, ^S^1232^R^, and ^R^1298^Q^) [18]. It was found that the Attenuated AJ1102-R strain mutated from D to E at position 1249 aa and showed five aa insertions at position 1267–1268 aa (^1280^AIVDV^1284^), which was consistent with the characteristics of the AJ1102 strain after 70 passages. Our results supported the suggestion that YNLP 2022 belongs to the GII clade, but its S protein was D at position 1249 aa, consistent with the classical attenuated vaccine strain CV777; whether the mutation related to the failure of vaccination with the variant attenuated vaccine remains to be examined later.

The tissue tropism of S protein did not change after INDEL, and it could still induce neutralizing antibodies. The S protein epitopes SS2 (^748^YSNIGVCK^755^) and 2C10 (^1368^GPRLQPY^1374^) can induce neutralizing antibodies against PEDV that are both conserved among wild-type strains in China [35], and the Epitopes COE (499–638 aa) and SS6 (^764^LQDGQVKI^771^) exhibit diversity among most wild-type strains, which is also a mutation-prone region in the epitope region [40,41]. Multiple amino acid positions were found to show mutations in the SS2, SS6, and COE epitopes. The variation of S gene epitope COE and SS6 may provide important reference for the development of attenuated vaccines. In addition, we note that the S protein of the YNBS 2022 strain has a unique change of five amino acids, and frequent variation in the S2 subunit may be the main reason for neutralization antibody failure. Further field epidemiological investigation showed that pigs infected with the YNBS 2022 strain were vaccinated with the attenuated vaccine of the strain AJ1102, which may be related to the S protein mutation of the YNBS 2022 strain.

Compared with M and N proteins, the structural protein S and accessory protein ORF3 were subjected to higher selection pressure, with a mutation rate of 57.1–71.4% [42]. S protein is not the only factor that affects the virulence of the virus. ORF3 also plays a role in enhancing the virulence of the virus. In contrast to the severely infected BJ2011C strain, the CHM2013-SBJ strain lacking 70 aa in ORF3, the pathogenicity was declined substantially. The Attenuated DR13 strain lacks 16 aa in the ORF3 gene, making it a candidate vaccine against PEDV infection [43]. However, not all ORF3 truncations were effective in reducing pathogenicity. Strains 17GXCZ-1ORF3d and HN2021 with naturally truncated ORF3 were both lethally toxic compared to field strains with intact ORF3 [44]. In this study, we found the ORF3 of the PEDV YN2021 strain had a large number of amino acid deletions, identical with Attenuated CV777 and the cell-adapted strains, while a full-length ORF3 strain with an intact truncation may contribute to the reduction of pathogenicity in the field strain. The ORF3 protein contains a four-helix transmembrane domain, which were TMD1 (40–63 aa), TMD2 (74–97 aa), TMD3 (118–136 aa), and TMD4 (151–172 aa), respectively [45]. Furthermore, the 91 aa residues encoded by ORF3 of attenuated PEDV lack TMD3 and TMD4, of which the domains have been described as forming a tetrameric assembly, playing an important role in regulating potassium channel activity, and were associated with the virulence of PEDV [45]. Whether the mutation detected in Yunnan PEDV strains will affect the virulence of the strains is worthy of further study.

M protein is involved in the viral envelope, interacts with the viral nucleus, and plays an important role in viral assembly. In 2016, Zhi et al. reported that PEDV strains from Thailand and China have no amino acid INDEL in the M protein [46]. In addition, three amino acid substitutions (^E^13^Q^, ^V^42^A^, and ^A^214^S^) were detected in the M protein of the Polish strain (0100/4T, 25364/2) [47]. We found that the M protein of the four strains has no substitution at the positions 13 aa and 214 aa, identical to those of CV777 (^13^E, ^214^A). Meanwhile, there is one aa substitution (^V^42^A^) in the YNLP 2022 and YNBS 2022 strains. Studies have shown that the ^E^13^Q^ mutations alter the hydrophobicity of the N-terminus of M protein, thereby affecting its antigenic activity, while the mutations in other proteins do not affect the hydrophobicity of M protein [48].

N protein is the most abundant protein in virus particles and provides the structural basis for the helical nucleocapsid of virus genomes, induces neutralizing antibodies, and participates in many biological and immunological activities of viruses. Although N protein has remarkable characteristics, compared with S protein, there are fewer studies about N protein. N proteins are also commonly used as diagnostic and vaccine development targets. Initially, the N protein was considered to be highly conserved, but a recent study by Bayesian phylogenetic analysis found that the PEDV N gene evolved at a similar rate as the S gene, approximately 10^−4^ substitutions/site/year, showing that the N and S genes evolve with rapid evolutionary and higher genetic diversity [49]. Genetic diversity may lead to changes in the antigenicity of N protein and may cause problems in the detection and diagnosis of PEDV. NEP-D4 and NEP-D6 have been identified in N proteins, located at residues 18-133 aa and 252-262 aa, respectively [50]. In this study, the amino acid sequence of N protein was compared with the CV777 strain in NEP-D4, while the four Yunnan strains all had one aa mutation at position 84 aa (^G^84^A^). In NEP-D6, both the YNBS 2022 and Attenuated AJ1102-R strains differed at position 262 aa (^K^262^N^). In addition, some differences were observed between the GI genotype of classical PEDV strains and the GII genotype of PEDV strains, which may affect the antigenicity of N protein.

The genetic diversity of RNA viruses is mostly determined by their large population, caused by high mutation rates. Recombinations are common in many RNA viruses and are associated with host range expansion, virulence enhancement, host immunity evasion, and drug resistance. Recombination is a main way of exchanging genetic information, and higher-frequency variations naturally happen significantly among different RNA viruses [51,52,53]. Recombination plays a crucial role in the diversity and evolution of coronaviruses by producing new strains. Studies showing that variant strains are generated by recombinations demonstrated that AH2012 was a recombination from KNU-0802 and JS-2004-2 [54]. We analyzed the genome recombination of three Yunnan epidemic strains and found that YNLP 2022 is a recombination from both GII-b strain PEDV-7C and GII-a strain YN1, and the recombination region is in the range nt4994–7605. The YNBS 2022 strain is a recombination from GII-b subtype strain 17GXZC-1ORF3c and GII-a subtype strain PEDV-CHZ, and the recombinant region is in the range nt16399–22326. Therefore, it is essential that surveillance and investigation of pig populations is carried out regularly, promoting the development of vaccines against PEDV strains or new variants that may lead to future epidemiology. Our findings will improve the molecular epidemiological survey of PEDV in Yunnan Province, facilitating understanding of its latest genetic progress. To better understand the evolution and genetic variation of PEDV, an extensive epidemiological survey needs to be carried out in Yunnan for more pig herds.

## 5. Conclusions

In conclusion, we reported here the first whole-genomic analysis of PEDV in Yunnan, China. The homology and genetic evolutionary analysis showed that the Yunnan epidemic strain YN2021 belonged to GI-b, and YNLP 2022 and YNBS 2022 belonged to GII-b. Compared with the classical vaccine strain (CV777) and the variant attenuated vaccine strain (Attenuated AJ1102-R), the PEDV epidemic strains YN2021, YNLP 2022, and YNBS 2022 showed INDELs at different positions, and the antigenic neutralizing epitopes showed polymorphic variation. The recombination results indicated YNLP 2022 and YNBS 2022 had genome recombination. This study may shed new light on the current PEDV infections and pave the way toward further control of PEDV infections in Yunnan.

## Figures and Tables

**Table 1:** The positive rates of PEDV during February 2021–December 2023 in Yunnan.

Year	Sample Numbers	Sample Types			Positive Numbers	Positive Rate (%)
		Feces	Intestine	Blood		
2021	128	58	26	44	33	25.78
2022	84	32	22	30	12	14.28
2023	7	3	2	2	2	28.57
Total	219	93	50	76	47	21.46

**Table 2:** The length of porcine epidemic diarrhea virus (PEDV) genomes and the functional genes.

Strain	Length (bp)	ORF1ab (bp)	S (bp)	ORF3 (bp)	E (bp)	M (bp)	N (bp)
YN2021	27,953	20,320	4149	276	231	681	1326
YNLP2022	28,036	20,345	4158	675	231	681	1326
YNBS2022	28,031	20,345	4158	675	231	678	1326
AJ1102-R	28,042	20,345	4170	675	231	678	1326

**Table 3:** Single amino acid variations in PEDV S protein.

Strain	Amino Acid Variations Based on CV777						
	577	897	901	982	1249	1300	1301
CV777	Q	G	Q	P	D	E	Q
DR13	Q	G	Q	P	D	E	Q
Attenuated CV777	Q	G	Q	P	D	E	Q
Attenuated DR13	Q	G	Q	P	D	E	Q
AJ1102	Q	G	Q	P	D	E	Q
Attenuated AJ1102-R	Q	G	Q	P	E	E	Q
ZL29	Q	G	Q	P	D	E	Q
YN2021	R	G	Q	P	D	E	Q
YNLP 2022	Q	G	Q	P	D	E	Q
YNBS 2022	Q	R	H	A	E	Q	H

**Table 4:** Recombination analysis of the complete PEDV genome.

Strain	Detect Method	Recombination Signal Yes/No	*p*-Value
YN2021	RDP	−	None
	GENECONV	−	None
	Bootscan	−	None
	MaxChi	+	1.53 × 10^−2^
	Chimaera	−	None
	3Seq	+	2.161 × 10^−2^
	SiSscan	−	None
YNLP 2022	RDP	+	1.549 × 10^−18^
	GENECONV	+	1.961 × 10^−18^
	Bootscan	+	4.224 × 10^−12^
	MaxChi	+	1.445 × 10^−8^
	Chimaera	+	4.310 × 10^−9^
	3Seq	+	3.080 × 10^−26^
	SiSscan	+	3.106 × 10^−8^
YNBS 2022	RDP	+	7.149 × 10^−17^
	GENECONV	+	6.743 × 10^−10^
	Bootscan	−	None
	MaxChi	+	1.986 × 10^−9^
	Chimaera	+	1.270 × 10^−9^
	3Seq	+	1.439 × 10^−7^
	SiSscan	+	3.683 × 10^−15^

## Data Availability

Data associated with this article are included in the Supplemental Materials. Whole-genome sequences of PEDV isolates are available in the National Center for Biotechnology Information (NCBI). Accession numbers for individual isolates are listed in the Supplementary Materials (Table S2).

## Supplementary Materials

The following supporting information can be downloaded at: https://www.mdpi.com/article/10.3390/vetsci11110548/s1, Figure S1: Nucleotide homology of PEDV genome; Figure S2: Amino acid homology of PEDV S protein; Table S1: PEDV genome-wide amplification primer information; Table S2: Information about the reference PEDV strains used in this study.
