# Supplementary figures and images for: Whole-Genome Analysis of Porcine Epidemic Diarrhea Virus from Yunnan, China
Source: Vet Sci. 2024 Nov 6;11(11):548. doi: 10.3390/vetsci11110548 (PMC11599152; doi:10.3390/vetsci11110548)

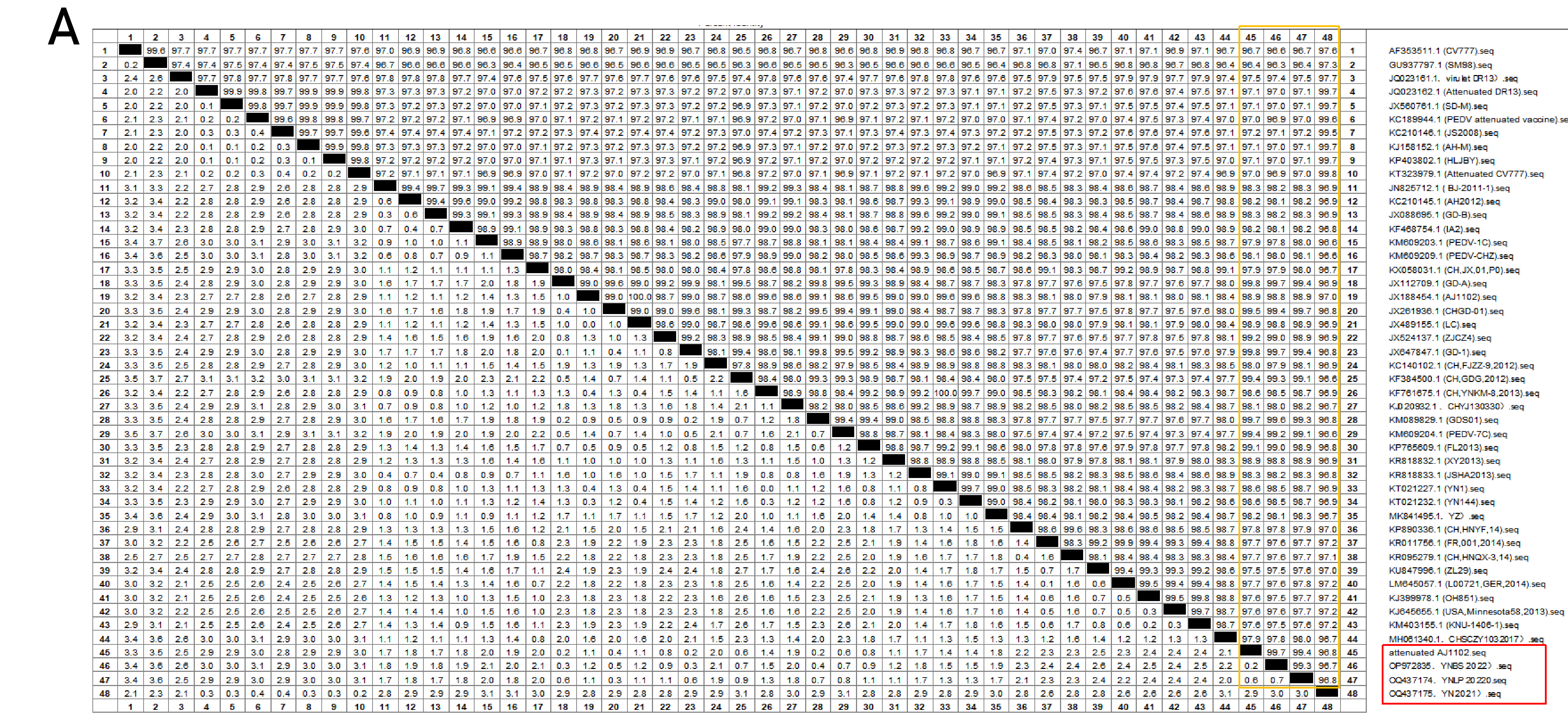

Supplement: Supplementary file 1 [file vetsci-11-00548-s001.zip › Supplementary File/Figure S1.jpg]

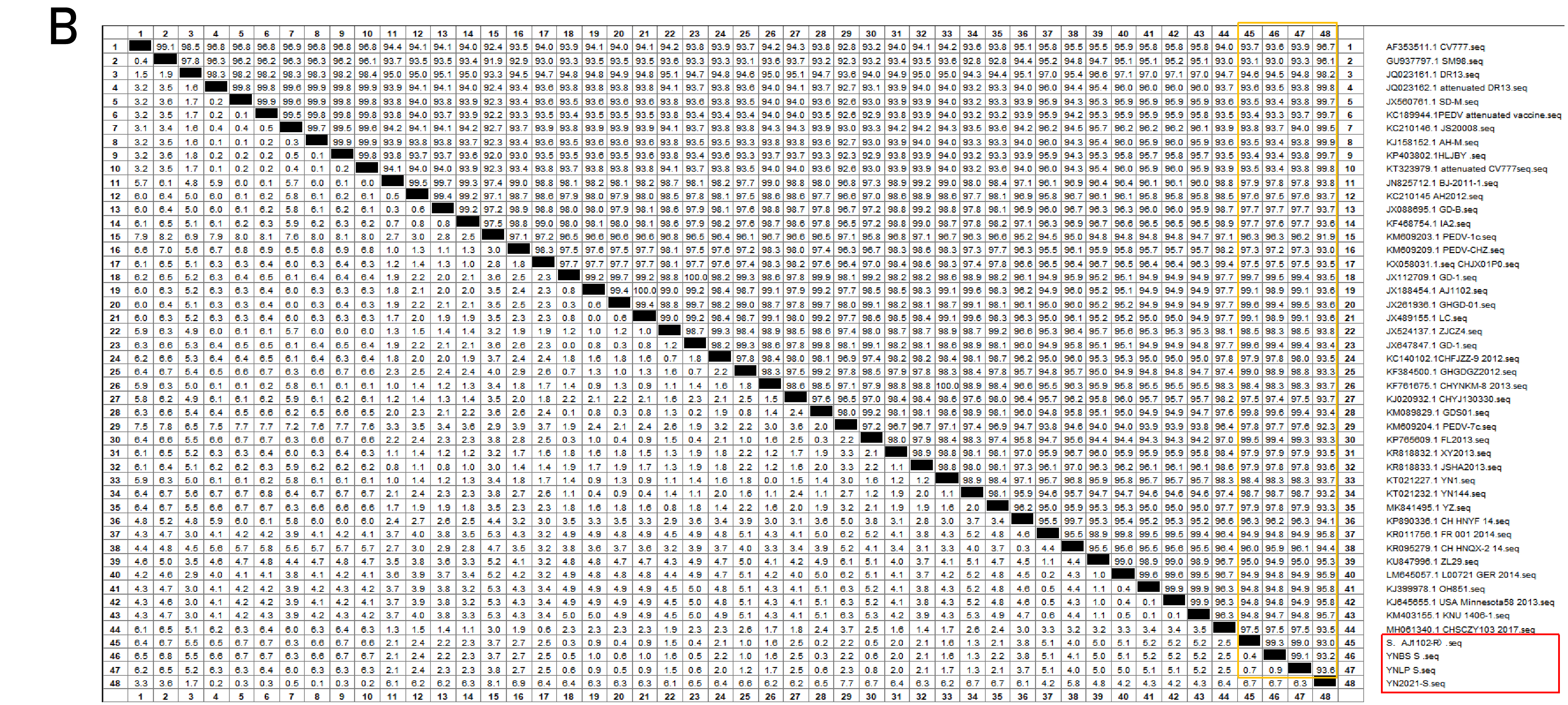

Supplement: Supplementary file 1 [file vetsci-11-00548-s001.zip › Supplementary File/Figure S2.jpg]
